# Toolkit for adapting community engagement studios to effectively engage older adults in research

**DOI:** 10.1017/cts.2025.66

**Published:** 2025-04-10

**Authors:** Shaye A. Kerper, Janelle C. Christensen, Steven M. Albert

**Affiliations:** University of Pittsburgh, Pittsburgh, PA, USA

**Keywords:** Older adults, community engagement, adaptation, toolkit, health promotion

## Abstract

Older adults have largely been excluded from health research despite bearing a disproportionate disease burden. The Community Engagement Studio (CES) model, initially developed at Vanderbilt University in 2009, allows potential research participants to help shape research to promote greater inclusion. The University of Pittsburgh adapted the CES model for older adults (OA-CES). Tailored specifically to older adults, OA-CES addresses underrepresentation in research by gathering valuable feedback that allows investigators to make research more accessible and relevant to older people. An OA-CES toolkit will help in adapting the model in other research areas to close the gap in research inclusion.

## Introduction and background

Research and public health initiatives are often designed with a strong focus on ensuring the health and safety of children and younger populations, leading to tailored interventions that address their unique needs. However, similar considerations for older adults are frequently overlooked, even though aging impacts metabolism, daily functioning, access to care, and overall well-being [1–5]. This lack of inclusion can lead to gaps in healthcare, research, and policy decisions that fail to address the diverse needs of older adults [6]. As a result, there is an urgent need to prioritize and expand research and policy efforts that cater to the aging population to ensure more equitable and effective healthcare outcomes.

A notable example of the risks associated with overlooking older adults in research is Benoxaprofen, a nonsteroidal anti-inflammatory drug released in the UK in 1980 [7]. Despite being primarily prescribed to older adults, the drug was tested on seventeen people aged 21–55 years [8]. Within two years of its release, it was linked to over 61 deaths, mostly among older patients [9]. This case underscores the dangers of failing to consider age-related differences in research and the urgent need for greater inclusion of older adults across studies. [10].

Addressing this gap requires research approaches that actively involve older adults, ensuring their perspectives shape study designs and healthcare decisions. Traditional methods of community engagement often fail to accommodate the specific challenges older adults face, such as mobility limitations, accessibility concerns, and technology barriers. Without targeted strategies, research efforts risk overlooking critical insights from this population.

One established approach for integrating community perspectives into research is the Community Engagement Studio (CES) model, which facilitates structured discussions between researchers and community members. While CES has been widely implemented across various populations, adaptations tailored to older adults are needed to address participation barriers effectively. In response to this need, we developed the Older Adult Community Engagement Studio (OA-CES), a modified version of CES designed to enhance accessibility and inclusion for older participants. This paper outlines the development of OA-CES and presents a structured toolkit to guide researchers in implementing this model.

## Community engagement studios and OA-CES adaptation

The CES model was initially developed at Vanderbilt University in 2009 to bring community members directly into the research process [11]. It provides structured, one-time forums where researchers present their projects, and community experts provide feedback that can shape study design, recruitment strategies, and outcome measures [12]. The CES is **not** intended to be a community advisory board nor to serve as a focus group; it provides a structured opportunity to gain community insight on study designs and has the potential to transform the way community members and research investigators work together [13,14].

Over time, the CES model has since been adapted by several institutions, including the University of Pittsburgh’s Clinical and Translational Science Institute (CTSI, a CTSA Hub) and the University of Pittsburgh (UPitt) Pepper Center, which specifically adapted the model to focus on older adults in the OA-CES [15,16].

At the University of Pittsburgh, the CTSI and the UPitt Pepper Center have implemented CES. CTSI recruits community experts through personal and professional networks, with a participant repository in development for future studies. Researchers access the CES model through a web-based platform, where they receive guidance on structuring their sessions and making their materials accessible to community members. Sessions are typically conducted virtually via Zoom, with transcripts and notes provided to researchers for integration into study designs.

The UPitt Pepper Center OA-CES further refines this approach by focusing specifically on the inclusion of older adults. Community experts undergo an orientation covering research fundamentals, the historical exclusion of older adults, and ethical considerations [17]. Recruitment efforts emphasize diversity by utilizing the Pepper Community Registry, which includes individuals aged 60+ living in the community, and the Platinum Senior Living Registry, which includes individuals aged 55+ residing in partnered senior living facilities, ensuring representation across age, race, location, and health conditions [16]. Research teams working with the OA-CES receive tailored support, including pre-session consultations to enhance the accessibility of their presentations. Sessions can be conducted in person or virtually, with structured feedback provided through notes, anonymized transcripts, and participant surveys. These adaptations ensure that older adults’ perspectives meaningfully inform research while addressing barriers to participation.

Table 1 outlines critical modifications in OA-CES that address older adults’ specific needs. Unlike the traditional CES model, OA-CES integrates accessibility supports (e.g., technology training, transportation assistance) and structured participant orientation to enhance engagement. These adaptations ensure that older adults’ insights shape research while minimizing barriers to participation.

**Table 1.** Comparison table of adaptation of CES

	Meharry-Vanderbilt Community-Engaged Research Core (Original)	Community Partners Community Engagement Studio (CTSI CES)	Pepper Center Older Adult Community Engagement Studio (OA-CES)
Time from Request for CES to Implementation	Two to four weeks are needed for the recruitment process.	Four weeks is standard, but the timeline is flexible based on the urgency or time required to complete the pre-CES materials.	In-person meetings: 4 weeks. Virtual meetings: 2 weeks.
Recruitment of Community Experts	Community experts are identified through community organizations and clinical practice – the panel consists of individuals who represent the researcher’s population of interest.	Community experts are recruited through convenience sampling, including personal contacts, professional contacts, and past participants. A participant registry is in the works.	The Pepper Community Research Registry and the Platinum Senior Living Research Registry.
Orientation/ Training of Community Experts	Project pre-meeting between the Community Engagement (CE) Studio staff and stakeholders.	No training of community experts.	Two-hour group orientation to “What is a Community Engagement Studio” and an adapted Community Partner Research Ethics Training (CEPRET) Training. Community experts were paid $50 for the training.
Recruitment of PI/ Research Teams for CES	Initiated by PIs and the research team request a CE Studio at any project stage.	Initiated by PIs and research teams request a consultation regarding CTSI services/CES.	Invitations are sent to Pepper and CTSI pilot grant recipients who want to engage more OA in research.
Orientation and Consultation with PI/ Research Teams	The researcher meets with the CE Studio team to clarify the studio’s focus, determine the stakeholder panel’s characteristics, and formulate the questions that will be posed to the stakeholders. The CE Studio staff also coaches the investigator on communicating effectively with non-researchers.	During the consultation, researchers can learn about studios in terms of concept and detail. Should they decide to have one, they will be provided with a template and a question guide to help them form their studio content. Once completed, these are reviewed, and notes are given for possible revisions to increase accessibility for general community members.	The Pepper Team meets with the PI to review their draft presentation slides one to two weeks before the event. The team provides an overview of the structure and what to expect and suggests ways to make the presentation more accessible and specific for the OA Community Experts.
Format of CES	Two-hour face-to-face event CE Studio (facilitated by a neutral moderator trained to ensure that the stakeholders are comfortable sharing their experiences and opinions).	Virtual of ZOOM, in-person based on PI request.	Virtual of ZOOM, in-person based on PI request.
Dissemination of CES Materials to Community Experts	If applicable, materials are brought to the in-person session.	If applicable, materials are distributed via email prior to or during the CES in the ZOOM session chat.	Materials are mailed and emailed to Community experts that RSVP to attend the OA-CES.
Feedback from Experts/ Stakeholders to the Research Team	Written summary report prepared by moderator.	Written and verbal feedback is provided by the CTSI team immediately after the CES. Community experts provide feedback via a survey link. A ZOOM audio transcript is provided.	Written feedback is provided by the moderator and community experts. A recording and de-identified transcript are provided after the CES.
Review Process (Researcher Feedback to Team)	A paper evaluation form is filled out.	Verbal feedback to the CTSI team.	An adapted online survey of the Vanderbilt post-CES survey is sent via email.
Payment Methods	Community Experts are paid $50 ($25/hour) for each CE Studio in which they participate.	Community Experts are paid $50 ($25/hour) for each CES in which they participate.	Community Experts are paid $50 ($25/hour) for each OA CES they participate in. An additional $25 is given for in-person events to cover travel costs.


CE, Community Engagement; CES, Community Engagement Studio; CPRET, Community Partner Research Ethics Training; CTSI, Clinical and Translational Science; OA; Older Adult; PI, Principal Investigator.

## Barriers and challenges in implementing OA-CES

In developing the OA-CES model, we encountered several barriers that influenced the structure of our toolkit, particularly in the areas of transportation, accessibility, and technology. Many older adults faced challenges with mobility and transportation, making it difficult to attend in-person sessions. To address this, we implemented transportation reimbursements, selected accessible venues, and provided pre-paid parking options. Similarly, accessibility within meeting spaces was a concern, requiring careful selection of locations with ramps, elevators, and seating accommodations.

For virtual participation, technology presented a significant barrier, as some older adults were unfamiliar with video conferencing platforms. To support engagement, we provided technical training, step-by-step guides, and real-time assistance during sessions. These challenges underscored the importance of proactive planning and informed the development of our toolkit, ensuring that older adults could fully participate in research without logistical or technological barriers.

## Toolkit for replicating OA-CES:

To replicate the OA-CES model, we propose a toolkit outlining critical strategies for recruitment, training, session format, feedback collection, and logistical support. This toolkit guides researchers and institutions in engaging older adults in their studies and can be adapted for use with diverse populations to address various research challenges.

### Recruitment strategies

- **Diverse Sampling:** Recruitment should prioritize diversity in age, race, gender, and location. Use community registries, outreach programs, and local networks to identify participants. Engage organizations that work with older adults, such as senior centers, retirement communities, and advocacy groups. While the primary goal is to engage older adults, sessions may include other stakeholders or experts when relevant. The balance between older adults and other experts should align with the study’s needs, ensuring that older adult voices remain central.
- **Tailored Outreach:** Different populations respond to different outreach methods. To reach older adults, use direct mail, phone calls, email, and community events. Mailed invitations should be followed by a phone call a week later, referencing the mail. For those with limited tech access, prioritize phone or in-person strategies.

### Training and orientation

- **Comprehensive Training:** Offer an initial training session introducing participants to research processes, ethics, and CES goals. Tailor the training to older adults, ensuring accessible materials and sessions are paced appropriately.
- **Technical Training and Accessibility:** Many older adults are unfamiliar with virtual participation tools, so training should include step-by-step guidance on accessing and using Zoom. This can be provided through:
  - One-on-one phone or in-person support before the session.
  - Printed guides with simple, visual instructions.
  - Short pre-session practice meetings for participants needing extra assistance.
  - A designated tech support staff member available before and during virtual sessions to troubleshoot issues in real time.

Accessibility should be prioritized across all sessions. This includes:

- Providing large-print materials and captions for virtual meetings.
- Ensuring in-person venues are wheelchair accessible with adequate seating and audio support.
- Allowing participants to contribute via phone if they lack internet access

### Session format

- **Flexible Participation Options:** Offer in-person and virtual sessions to accommodate different needs. In-person sessions should be in accessible locations with transportation support and refreshments. For virtual sessions, ensure participants have devices, stable internet, and tech support, maximizing engagement and allowing full participation.
- **Moderator:** Use a trained moderator to lead discussions, ensuring all participants have a chance to contribute. The moderator should actively facilitate an inclusive discussion where all older adults, as the key target population, can share their insights. While other experts may be present, the focus remains on amplifying older adults’ perspectives.

### Feedback mechanisms

- **Structured Feedback Forms:** Provide feedback forms before and after each session, allowing community experts to share thoughts. Forms can be mailed for in-person sessions or completed electronically for virtual ones.

### Logistical support

- **Transportation Assistance:** Provide transportation or reimburse travel expenses for in-person sessions. Ensure venues are accessible to those with mobility challenges.
- **Technology Support:** Ensure participants have the necessary devices and internet access for virtual sessions. Offer ongoing technical assistance, including one-on-one coaching, if needed.
- **Compensation:** Provide compensation for participants’ time and contributions, including financial compensation, covering travel costs, or providing meals during in-person sessions.

## Conclusion

In implementing the OA-CES, we faced several challenges related to transportation, accessibility, mobility, and technology usage. These barriers highlighted the importance of tailoring solutions to meet the specific needs of older adults, ensuring their full participation in the research process. To address transportation barriers, we provided pre-paid parking passes, transportation reimbursements, and selected venues near public transit and rideshare services. Accessibility concerns were mitigated by ensuring venues had wheelchair-accessible entrances, ramps, restrooms, and adequate seating, fostering a welcoming and inclusive environment.

By addressing these barriers, we empowered community experts to focus on sharing their experiences and insights, thereby enriching the research process. Removing obstacles related to transportation, accessibility, and technology-facilitated participation not only strengthened the connection between community members and research teams but also enhanced the quality of feedback received. These efforts emphasize the necessity of fostering research environments that prioritize inclusivity and accessibility, ensuring that older adults can actively contribute to shaping research outcomes. The solutions we developed are not just a one-time fix but a model for future research aiming to engage older adults effectively. These strategies underscore the importance of including older adult voices in research, ensuring that studies are more inclusive and reflective of the unique challenges older adults face, thus enhancing the relevance and impact of research outcomes. Through these efforts, we laid the groundwork for a more equitable and inclusive research environment that can be adapted for diverse populations. The author’s (SAK) master’s thesis focuses on feedback from 13 researchers who participated in an OA-CES using the toolkit strategies outlined above, highlighting the necessity of obtaining feedback from the targeted research group, in this case older adults. [18]. Some of these adaptations may also be useful for other groups, making this paper a valuable resource for researchers looking to implement CES with different populations.

As research and public health efforts continue to evolve, integrating engagement models that address the unique needs of older adults and other underrepresented populations is critical. OA-CES represents a meaningful step toward a more equitable and inclusive research landscape, offering a scalable approach that can be refined and applied across various community settings. Future studies can further explore and tailor these engagement strategies to specific demographic groups, reinforcing the broader impact of participatory research methods in ensuring that all voices are heard and valued in the research process.

## References

[1] Khan MZ , Munir MB , Khan SU , et al. Representation of women, older patients, ethnic, and racial minorities in trials of atrial fibrillation. Pacing Clinical Electrophis. 2021;44(3):423–431. doi: 10.1111/pace.14178. PMC8404149 33512027

[2] Bourgeois FT , Orenstein L , Ballakur S , Mandl KD , Ioannidis JPA. Exclusion of elderly people from randomized clinical trials of drugs for ischemic heart disease. J Am Geriatr Soc. 2017;65(11):2354–2361. doi: 10.1111/jgs.14833. 28306144 PMC5601009

[3] Kalyani RR , Golden SH , Cefalu WT. Diabetes and aging: unique considerations and goals of care. Diabetes Care. 2017;40(4):440–443. doi: 10.2337/dci17-0005. 28325794 PMC5360288

[4] Helfand BKI , Webb M , Gartaganis SL , Fuller L , Kwon CS , Inouye SK. The exclusion of older persons from vaccine and treatment trials for coronavirus disease 2019—Missing the target. JAMA Intern Med. 2020;180(11):1546. doi: 10.1001/jamainternmed.2020.5084. 32986099 PMC7522773

[5] Lockett J , Sauma S , Radziszewska B , Bernard MA. Adequacy of inclusion of older adults in NIH-funded phase III clinical trials. J Am Geriatr Soc. 2019;67(2):218–222. doi: 10.1111/jgs.15786. 30693958

[6] Henry C. Mechanisms of changes in basal metabolism during ageing. Eur J Clin Nutr. 2000;54(S3):S77–S91. doi: 10.1038/sj.ejcn.1601029. 11041079

[7] Abraham J. Scientific standards and institutional interests: carcinogenic risk assessment of Benoxaprofen in the UK and US. Soc Stud Sci. 1993;23(3):387–444. doi: 10.1177/0306312793023003001.

[8] Smith GL , Goulbourn RA , Burt RA , Chatfield DH. Preliminary studies of absorption and excretion of benoxaprofen in man. Br J Clin Pharmacol. 1977;4(5):585–590. doi: 10.1111/j.1365-2125.1977.tb00790.x. 303115 PMC1429149

[9] Lueck TJ. AT LILLY, THE SIDE-EFFECTS OF ORAFLEX. The New York Times. August 15, 1982. (https://www.nytimes.com/1982/08/15/business/at-lilly-the-side-effects-of-oraflex.html). Accessed June 3, 2024.

[10] Liu Q , Schwartz JB , Slattum PW , et al. Roadmap to 2030 for drug evaluation in older adults. Clin Pharma and Therapeutics. 2022;112(2):210–223. doi: 10.1002/cpt.2452. 34656074

[11] Joosten YA , Israel TL , Head A , et al. Enhancing translational researchers’ ability to collaborate with community stakeholders: lessons from the community engagement studio. J Clin Trans Sci. 2018;2(4):201–207. doi: 10.1017/cts.2018.323. PMC6382358 30820357

[12] Joosten YA , Israel TL , Williams NA , et al. Community engagement studios: a structured approach to obtaining meaningful input from stakeholders to inform research. Acad Med. 2015;90(12):1646–1650. doi: 10.1097/ACM.0000000000000794. 26107879 PMC4654264

[13] Israel Tiffany , Farrow Helena , Joosten Yvonne , Vaughn Yolanda. Community Engagement Studio Toolkit 2.0 | Meharry-Vanderbilt Alliance. (https://www.meharry-vanderbilt.org/community-engagement-studio-toolkit-20). Accessed August 21, 2023.

[14] Meharry-Vanderbilt Community Engaged Research Core – VICTR – Vanderbilt Institute for Clinical and Translational Research. (https://victr.vumc.org/meharry-vanderbilt-community-engaged-research-core/). Accessed June 3, 2024.

[15] Community PARTners. (https://ctsi.pitt.edu/research-services/core-services/community-partners/). Accessed May 31, 2024.

[16] Pittsburgh Pepper Center - University of Pittsburgh. (https://www.pepper.pitt.edu/). Accessed November 11, 2023.

[17] Yonas MA, Jaime MC , Barone J , et al. Community partnered research ethics training in practice: a collaborative approach to certification. J Empir Res Hum Res. 2016;11(2):97–105. doi: 10.1177/155626461665080. PMC4917430 27241871

[18] Kerper SA. Refining Research through Dialogue: Lessons from Older Adult Community Engagement Studio Feedback [master’s thesis]. Pittsburgh, PA: University of Pittsburgh; 2024. (https://d-scholarship.pitt.edu/45824/1/Kerper%20MPH%20Essay%202024.pdf). Accessed February 24, 2025.

